# Exploring Lifestyles and Sensory Processing Patterns of Toddlers in Relation to Sleep Patterns Using Body Movement Analysis

**DOI:** 10.3390/clockssleep7020025

**Published:** 2025-05-15

**Authors:** Azusa Ono, Yoshiko Iwatani, Arika Yoshizaki, Tomoko Nishimura, Ikuko Mohri, Kuriko Kagitani-Shimono, Masako Taniike

**Affiliations:** 1 Department of Child Development, United Graduate School of Child Development, Osaka University, Osaka 565-0871, Japan; ono.azusa.7h8@osaka-u.ac.jp (A.O.); ymorita@ped.med.osaka-u.ac.jp (Y.I.); nishimura.ugscd@osaka-u.ac.jp (T.N.); mohri.ugscd@osaka-u.ac.jp (I.M.); kuriko@ped.med.osaka-u.ac.jp (K.K.-S.); 2 Molecular Research Center for Children’s Mental Development, United Graduate School of Child Development, Osaka University, Osaka 565-0871, Japan; arika@kokoro.med.osaka-u.ac.jp

**Keywords:** sleep quality, sleep habits, toddler, body movement during sleep, sensory processing patterns

## Abstract

This study explored the impact of lifestyle habits and sensory processing patterns on sleep quality by analyzing body movements (BMs) during the first and last 3 h of sleep in toddlers. We collected cross-sectional data about sleep-related habits from 58 toddlers using a mobile application. Actigraphy measured BMs during nighttime sleep and 1 h before bedtime, as well as sleep latency, over 8 consecutive days. The Infant/Toddler Sensory Profile was used to assess the toddlers’ sensory processing patterns. The participants had a mean age of 22.0 ± 2.0 months. BMs were significantly lower during the first 3 h of sleep. Longer sleep latency was significantly associated with media use and higher activity levels before bedtime. Ending a nap earlier and consuming a substantial breakfast were correlated with lower BMs during the first 3 h of sleep. Auditory and oral sensory scores were positively correlated with BMs during the first 3 h of sleep. However, no specific factors related to lifestyle habits or sensory processing patterns were found to correlate with BMs during the last 3 h of sleep. Lifestyle habits and sensory processing patterns have a significant impact on toddlers’ sleep quality, emphasizing the importance of appropriate routines and environments.

## 1. Introduction

It is important to note that poor sleep quality significantly impacts an adult’s daily life. Sleep is essential for various brain functions, including memory, learning, and emotional regulation [1,2,3]. Poor sleep quality is associated with negative physical and mental health outcomes, such as compromised immune function, cognitive decline, and behavioral issues [4,5].

Sleep is also crucial for children’s health, development, and overall well-being. Particularly in early childhood, sleep patterns change rapidly. A decrease in total sleep time and a gradual reduction in daytime sleep, leading to the integration of sleep into a single nighttime period, are commonly observed during this stage [6]. Young children with inadequate sleep experience a range of mental and behavioral issues, including hyperactivity, inattention, impulsivity, and emotional challenges [7,8,9,10,11,12,13].

The sleep problems of children, such as difficulty settling into sleep (including bedtime resistance), difficulty in falling asleep, or nighttime awakenings, affect parents’ mental health and contribute to parental stress [14,15]. Such sleep-related concerns may impede the formation of healthy attachments between parents and children [16]. Therefore, a sleep environment that prevents such sleep problems in children is essential for supporting caring families.

Several studies have demonstrated that daily habits contribute to poor sleep quality. For instance, prolonged screen time and late-night media usage are associated with reduced sleep duration in preschoolers [17]. Engagement in other stimulating activities before bedtime also negatively impacts sleep quality [18]. Moreover, insufficient daytime physical activity or excessive activity before bedtime can lead to increased nocturnal awakenings and shorter sleep duration [19]. Our research group reported that a well-structured daytime schedule for toddlers correlates with improved sleep quality [20].

Also, differences in sensory processing patterns, which refer to over- or under-reactivity to sensory input, may significantly impact children’s sleep. The Sensory Profile, which evaluates sensory processing patterns across tactile, oral, auditory, vestibular, and visual sensory domains, is a widely used tool in research and clinical practice. Epidemiological studies suggest that 5–16% of children exhibit sensory abnormalities that significantly affect their daily lives [21,22]. Children with sleep problems frequently demonstrate sensory difficulties across all domains [23,24]. The processing of sensory information varies according to the stages of sleep, influencing sleep–wake regulation [25]. As a result of heightened arousal from environmental stimuli, children with oversensitivity may experience difficulties falling or staying asleep, thereby compromising sleep quality [26].

Lifestyle habits and sensory abnormalities can disrupt sleep. However, there is limited research specifically focusing on the quality of toddlers’ sleep, and many available reports primarily rely on parent reports of sleep, lacking objective indicators [27].

Assessing body movements (BMs) is an important indicator of sleep quality. Typically, BMs are lowest during deep sleep and increase during light sleep, REM sleep, and waking, in that order [28,29]. A higher proportion of deep sleep is one of the indicators of better sleep quality; therefore, increased BMs are indicative of poorer sleep quality [30].

Actigraphy, which is widely used in sleep research, quantifies BMs and offers a reliable means of assessing sleep [31,32]. It acquires objective data noninvasively within an individual’s usual environment, unlike polysomnography, which is conducted in laboratory settings outside the home. In previous actigraphy studies, algorithms and software-estimated sleep parameters, such as total sleep time, sleep efficiency, wakefulness after sleep onset, and sleep latency, have been used [31]. However, particularly in toddlers, it has been challenging to assess sleep quality based on sleep efficiency because of poor specificity in detecting wakefulness after sleep onset across various devices. Although the algorithm used in this actigraphy has been validated against polysomnography in adults, and a strong correlation has been confirmed between parental observations and actigraphy data for sleep latency [33], previous research indicates that actigraphy is generally more accurate in detecting sleep onset than in identifying nocturnal awakenings in toddlers when age-specific algorithms are lacking [34]. Therefore, based on these considerations, we focused on the level of BMs and sleep latency to evaluate sleep quality.

This study aimed to investigate the relationship between toddlers’ sleep quality, lifestyle habits, and sensory processing patterns using actigraphy. Although numerous studies have examined the relationship between young children’s lifestyle habits and sleep duration or timing, few have focused on sleep quality. By incorporating actigraphy-based body movement analysis, our approach provides an objective assessment of sleep quality, reducing reliance on parental reports. This study offers new insights into early childhood sleep and contributes to the promotion of better health and well-being for toddlers and their caregivers.

## 2. Results

Table 1 summarizes the mean ± standard deviation (SD) values for demographic data, sleep parameters, BMs during sleep, sensory processing scores, and other essential metrics. This study included 30 boys and 28 girls, with an average age of 22.0 ± 2.0 months. The average sleep onset time was 9:24 PM (SD 0:48), and the average sleep end time was 6:49 AM (SD 0:40). Total sleep time during the night was 564.8 ± 43.9 min. Sleep latency, defined as the interval between bedtime and sleep onset, was 33.3 ± 16.3 min. The average net sleep time measured by actigraphy, defined as total sleep time excluding periods identified as wakefulness, was 397.6 ± 65.1 min. Based on this, the calculated average sleep efficiency was 64.5 ± 8.5%. However, it should be noted that these values were derived using an algorithm developed for adults and may not accurately reflect sleep patterns in young children. Concerning lifestyle habits, 42 participants (72.4%) reported consuming a substantial breakfast, whereas 30 participants (51.7%) indicated that they played outside in the morning. Significant variability was observed in nap end times and daily screen times, with the average nap end time being 3:17 PM (SD 1:08), and the mean daily screen time, including activities such as watching videos or using mobile phones, recorded at 92.0 ± 69.0 min. Ten participants (17.2%) reported engaging in screen time of 1 h before bedtime.

**Figure 1:**
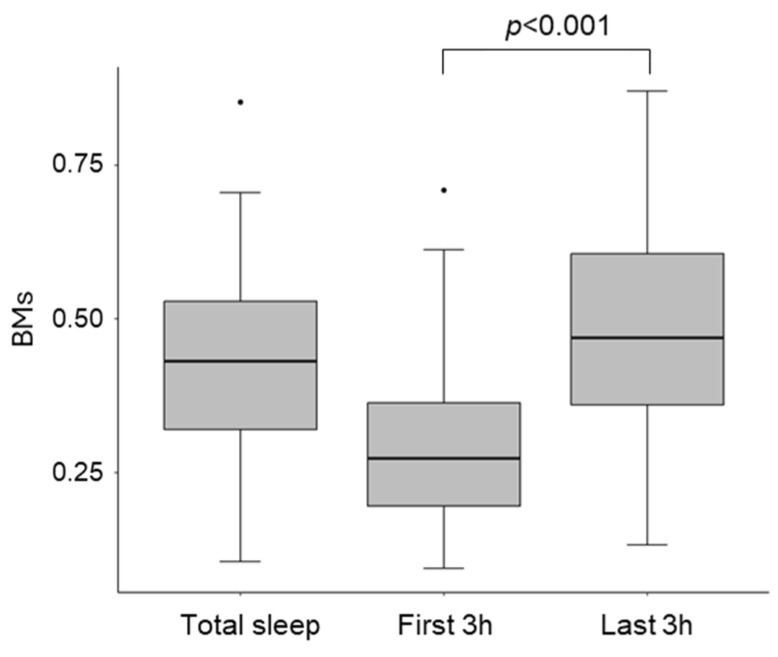
The distribution of BMs during total sleep, the first 3 h, and the last 3 h of sleep. BMs were significantly lower in the first 3 h compared to the last 3 h, reflecting reduced activity during the deep sleep phase.

BMs during sleep were categorized into three groups: BMs during overnight sleep (total sleep BMs), BMs during the first 3 h of sleep from sleep onset (first 3 h BMs), and BMs during the last 3 h of sleep until the end of sleep (last 3 h BMs). The first 3 h BMs were significantly lower than the last 3 h BMs (t = −6.93, df = 114, *p* < 0.001; Figure 1).

The average sensory processing scores for all subdomains were within the normal range [35]. Of the 58 participants, 9 (16%) had tactile, 12 (21%) had oral, 9 (16%) had auditory, 10 (17%) had vestibular, and 10 (17%) had visual sensory processing scores outside the normal range.

**Figure 2:**
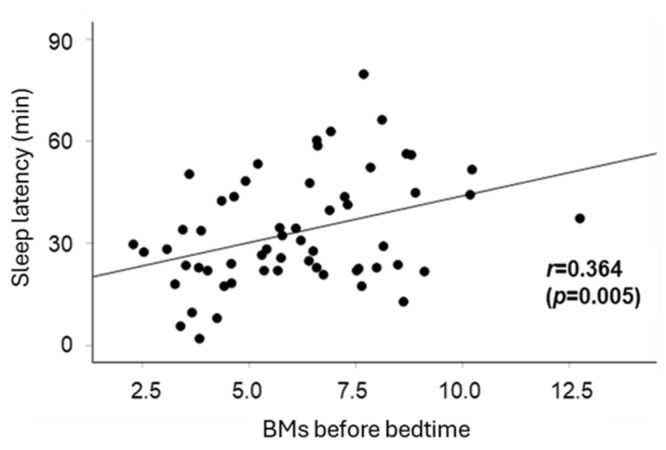
The correlation between sleep latency and BMs before bedtime. The correlation coefficient, *r* = 0.364 (*p* = 0.005), indicated a statistically significant and moderately positive correlation between BMs before bedtime and sleep latency. The line in the scatter plot represents a regression line based on the estimated values derived from simple linear regression analysis. The points on the scatter plot correspond to the actual measured values.

Next, factors contributing to extended sleep latency were examined (Table 2). Sleep latency was positively correlated with screen time before bedtime (*p* = 0.016) and BMs (*p* = 0.003). Figure 2 illustrates the relationship between BMs before bedtime and sleep latency, and a moderate positive correlation was observed (r = 0.364, *p* = 0.005).

In analyzing the relationship between BMs during sleep and lifestyle habits, the first 3 h BMs were significantly negatively correlated with consuming a substantial breakfast (*p* = 0.009) and the end time of napping before 3 PM (*p* = 0.046), as presented in Table 3. However, the presence or absence of screen time and BMs before bedtime were not significantly associated with the first 3 h BMs. In contrast, no lifestyle habits demonstrated any associations with total sleep BMs or the last 3 h BMs (Table 3). In addition, a multiple linear regression analysis was conducted to examine the associations between sleep efficiency and lifestyle habits, using the same predictor variables as in Table 3. Sleep efficiency was positively correlated with consuming a substantial breakfast (coefficient = 5.87, 95% CI: 0.76 to 11.0, *p* = 0.025). No other lifestyle factors showed significant associations with sleep efficiency.

**Figure 3:**
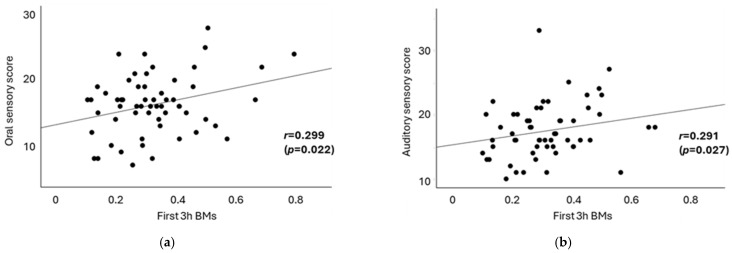
The correlation between the first 3 h BMs and sensory processing patterns. Shown are statistically significant relationships between the first 3 h BMs and oral sensory processing patterns (**a**) and auditory sensory processing patterns (**b**), with a Pearson correlation coefficient of *r* = 0.299 (*p* = 0.022), and with a Spearman correlation coefficient of rho = 0.291 (*p* = 0.027).

Table 4 presents the results of the correlation analysis between sensory profile scores and sleep latency, as well as between the first 3 h BMs and the last 3 h BMs. In terms of sensory processing, sleep latency was significantly associated only with visual sensory scores (r = −0.276, *p* = 0.036). A moderate positive correlation was observed between the first 3 h BMs and oral sensory scores (Pearson’s r = 0.299, *p* = 0.022; Figure 3a), as well as between the first 3 h BMs and auditory sensory scores (Spearman’s rho = 0.291, *p* = 0.027; Figure 3b). However, no significant correlations were identified between the last 3 h BMs and any sensory scores. These findings indicate that oral and auditory sensory abnormalities may contribute to poor sleep quality. The association between sensory processing scores and lifestyle habits was not observed in this study. In addition, no significant associations were found between sleep efficiency and sensory processing scores.

## 3. Discussion

This study assessed the relationship between sleep quality, as estimated by BMs, and lifestyle factors, as well as sensory processing patterns. Our study makes several novel contributions to the literature on toddler sleep. First, this study utilized a unique methodology to analyze the impact of lifestyle habits and sensory processing patterns on sleep by dividing it into early (first 3 h) and late (last 3 h) segments. The initial 3 h of sleep exhibited lower BMs, consistent with previous findings that deep sleep, which predominates during the early part of the night, is associated with reduced BMs [28,36].

The enrichment of N3 sleep is considered a key component of sleep functions, such as the consolidation of memories experienced during the previous wake period and the restoration of synaptic homeostasis [37,38]. Borbély’s two-process model of sleep regulation suggests that the sleep–wake-dependent homeostatic process (Process S) interacts with a process controlled by the circadian pacemaker (Process C) [39]. This model proposes that sleep commences when homeostatic sleep pressure, influenced by various factors, including circadian regulation, accumulates during wakefulness and reaches a sleep threshold. The representative aspect of Process S is the activity of slow waves during N3 sleep [40].

We discovered that earlier nap endings and higher breakfast consumption are associated with reduced first 3 h BMs (Table 3). It is plausible that ending naps earlier increases sleep pressure, which leads to improved sleep quality. There are two potential explanations for the relationship between the first 3 h BMs and substantial breakfast consumption. One possibility is that a substantial breakfast consumption results from sufficiently lowered sleep pressure, leading to refreshed wakefulness. Research indicates that children with insufficient sleep tend to have a lower rate of breakfast consumption [41,42]. Additionally, during infancy and early childhood, problematic eating behaviors have been linked to irregular sleep patterns and frequent nocturnal awakenings [43,44]. Another possibility is that breakfast consumption affects sleep pressure by resetting the circadian rhythm to a 24 h cycle, indicating an interaction between processes S and C. It has been reported that eating breakfast affects circadian rhythms by impacting the body’s internal clock through factors such as enhancing the thermogenic response, regulating endocrine factors, and expressing clock genes [45,46,47,48].

It has been suggested that sleep pressure is closely related to sleep onset latency, which refers to the time taken to fall asleep. Utilizing media later in the evening or in the bedroom decreases sleep pressure, which contributes to increased sleep latency, and as the wake-up time remains fixed, the duration of nighttime sleep is shorter [17,20]. We identified an association between media usage before bedtime and sleep latency (Table 2), but not with sleep quality as assessed by the first 3 h BMs. This inconsistency in the data between the first 3 h BMs and sleep latency may be explained by the model proposed by Murata et al. [20]. According to their model, the latent factor “sleep pressure”, defined by wake-up time, bedtime, nap end time, and daily activities, is negatively and indirectly influenced by the latent factor “hyperarousal”, characterized by late media usage or the total duration of media usage. Activities and media usage before bedtime appear to elevate the threshold for entering sleep, whereas sleep quality—especially during the early stages of sleep—may be influenced by other complex lifestyle factors, such as nap end time.

This study did not provide clear evidence of indicators associated with the last 3 h BMs. The last 3 h are predominantly composed of NREM light sleep and REM sleep. Consequently, the influence of sleep during this period was obscured by the interplay of different functions associated with these sleep stages. Future studies should evaluate the physiological significance of the last 3 h of sleep.

Another novel finding of this study is the positive correlation between the first 3 h BMs and scores on auditory and oral sensory processing patterns (Table 4 and Figure 3). Sensory input can influence sleep–wake rhythms [49]. Additionally, children with sensory sensitivities may experience hyperarousal, leading to increased BMs [25]. Regarding auditory sensory processing, individuals with insomnia are known to exhibit heightened responses to auditory stimuli across various stages of sleep [50]. This suggests that auditory hyperresponsiveness may interfere with the maintenance of sleep by increasing arousal levels. In terms of oral sensory processing patterns, oral health issues such as dental pain have been associated with sleep disturbances, indicating a link between conditions affecting the mouth and challenges in achieving quality sleep [51]. However, the connection between oral sensory processing patterns and sleep remains underexplored. These findings suggest that toddlers with increased sensory sensitivities may have difficulty maintaining deep sleep. Conversely, this study found that higher scores in visual sensory processing were associated with shorter sleep latency. Previous research has shown inconsistent results regarding the relationship between visual sensitivity and sleep quality or sleep latency, leaving this relationship unclear [23,26]. In this study, most participants lived in households where the bedroom environment was dark and visual stimuli were minimal, which may have mitigated any negative impact of visual input on sleep onset. We should also consider the possibility that short sleep onset latency, which may be caused by sleep deprivation, could affect sensory sensitivities. However, the mechanism underlying this finding remains unclear.

In summary, to promote smooth sleep onset and high-quality rest in toddlers, it is crucial to create a calm bedtime environment that minimizes excitement and sensory stimulation [18,52]. For example, implementing soundproofing to reduce noise and engaging in relaxing, calm activities before bedtime can significantly improve sleep quality and support overall well-being.

Our study had some limitations. First, the evaluation of sleep quality lacks direct EEG measurements, which potentially limits a comprehensive assessment of sleep stages. Although the reliable assessment of sleep onset latency ideally requires at least 14 days of monitoring [53,54,55,56], the high correlation between sleep onset latency reported by caregivers and that calculated using the actigraphy in this study was confirmed by our previous reports [33]. Second, daytime activity data were not collected using actigraphy because of the risk of device ingestion. Instead, we relied on caregiver reports for outdoor activity times. Third, in Japan, children’s bedtime is determined by the time the caregiver thinks the child should go to bed or the caregiver’s bedtime. Furthermore, children often exhibit bedtime resistance because they want to watch TV or for other reasons, and bedtime is often determined by parent–child interactions, resulting in the SD of bedtime being as long as 48 min, as shown in Table 1. Fourth, information on lifestyle habits relies exclusively on subjective reports from caregivers, which may introduce recall bias. However, this bias was minimized as the data were recorded daily using Nenne-Navi^®^. Lastly, there is a possibility that lifestyle habits and sleep quality may have influenced sensory traits, although the causal relationship remains unclear. This limitation should be addressed in future research to gain a better understanding of how these factors are related to sleep.

## 4. Materials and Methods

### 4.1. Participants

We recruited children aged 18–25 months from Osaka University through flyers, websites, social media, and other outreach methods from March 2021 to January 2023. The inclusion criteria required participants to be healthy, born full-term, and within the specified age range (18–25 months), and informed consent was obtained from caregivers for their participation. Additionally, recruited children needed to be capable of wearing the provided actigraphy devices.

Participants were excluded from this study if there were suspicions of child abuse or if they presented with any obvious neurodevelopmental disorders (e.g., abnormalities noted during a health checkup, previous usage of a child developmental support center, etc.).

**Figure 4:**
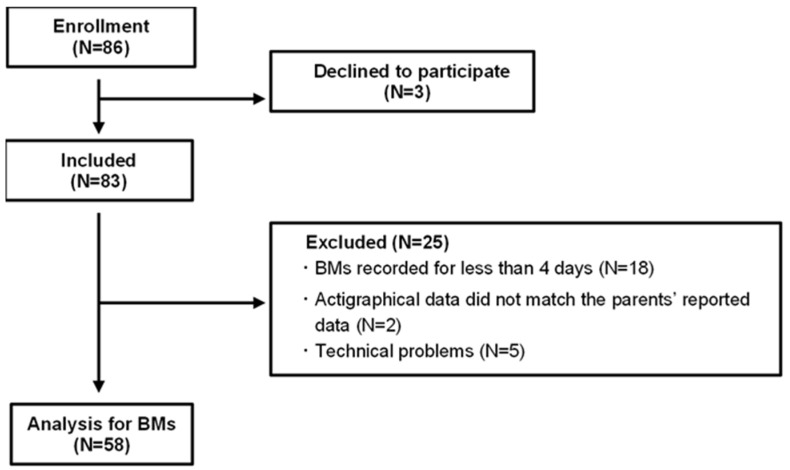
Flow diagram of this study.

After 3 children declined to participate, 83 children were enrolled in this study. Individuals for whom actigraphy data were recorded for less than 4 days because of atypical sleep conditions, such as colds, overnight stays, or forgetting to wear the devices, were excluded (N = 18). Furthermore, children whose monitored data did not align with the subjective data input by caregivers via the mobile application were excluded (N = 2) to maintain data consistency throughout this study. Technical issues with the actigraphy, such as water damage rendering it unusable and preventing data extraction, also resulted in exclusions (N = 5). Ultimately, the sample size was reduced to 58 (Figure 4). We confirmed the absence of signs of obstructive sleep apnea, restless legs syndrome, and NREM parasomnias from the caregivers of the included children.

### 4.2. Actigraphy

Children’s BMs were measured using an actigraph (MTB-220; Acos Co. Ltd., Chiba, Japan) over 1 week, during which sleep-related lifestyles were reported simultaneously. This actigraph is a small, lightweight (9 g), coin-shaped device (external dimensions: 27 mm in diameter and 9.8 mm in depth, including a clip) that records physical activity using an internal 3-axis accelerometer. Every 0.125 s, the system sums the number of times that the acceleration exceeds a reference value, recording it as the BM value in 2 min intervals. The BM intensity is calculated on a scale from 0 to 63 (64 levels), with zero indicating that the participant did not move and larger values indicating higher levels of activity. The BM scoring criteria of this actigraph against polysomnography have been previously validated [57]. Caregivers were instructed to attach the actigraph to their toddler’s clothing using the clip on the back of the device at all times, except during attendance at preschool or childcare facilities. The actigraph was modified to connect to a smartphone via Bluetooth instead of FeliCa, and caregivers were asked to transmit actigraph data through their smartphones to the server at Osaka University. The BM data retrieved from the actigraph were downloaded to a personal computer, and sleep onset latency was analyzed using SleepSignAct ver. 2 software (Kissei Comtec Co., Ltd., Nagano, Japan) [57,58]. The average BMs, overnight and during a specific daytime period (specifically, 1 h before bedtime), were manually calculated by a researcher (A.O.). The BM values recorded every 2 min were summed for each time period, and the average per minute was calculated.

In addition, activity levels were compared between the early and late stages of sleep, namely the first 3 h after sleep onset, which are rich in deep sleep stages, and the last 3 h before waking, which are rich in rapid eye sleep stages. These 3 h periods are expected to include at least two NREM-REM cycles.

### 4.3. Acquisition of Sleep-Related Habits and Sensory Processing Patterns

We utilized “Nenne Navi^®^” ver. 1.0.0 to gather data on the sleep-related habits of children. The details of Nenne Navi^®^, an interactive smartphone application designed to improve children’s sleep, have been previously reported [33]. Briefly, caregivers provided input regarding their toddlers for a duration of 8 consecutive days, which was transmitted via smartphone to the university server. The information collected on sleep-related habits included waking time, nap time, bedtime, screen time, outdoor playtime, meal time, bathing time, and sleep latency [20]. The data were analyzed, and various forms of practical and personalized advice were sent to each caregiver. For this study, only the sleep-related habits at baseline, prior to intervention, were analyzed.

Initially, we examined outdoor playtime in the morning to evaluate daytime physical activity. We specified “the morning” since late activities may disrupt the wake–sleep circadian rhythm. Information regarding the end time of naps and the conclusion of media use, including television, tablets, and mobile phones, was analyzed. The breakfast consumption of children was selected as an indicator of their mood and refreshment upon waking. In these analyses, we categorized groups based on whether the habit in question occurred for more than half of the week or less than half of the week. Additionally, we assessed activities conducted before bedtime in terms of their intensity, as well as the amount of media consumption based on their duration.

Sensory processing patterns were measured using the Infant/Toddler Sensory Profile questionnaire, which is a parent-report tool that assesses children’s sensory reactions or behavioral responses to sensory stimuli and includes 48 items. The questionnaire domains are scored across six sensory modalities (auditory, visual, tactile, vestibular, oral, and multisensory) that reflect basic sensory responses in young children. The original version of the questionnaire was developed and validated using standardized data from children aged 7 to 36 months [59]. The Japanese version has also been adapted and validated for this age group, demonstrating high internal consistency and construct validity through standardization studies [35]. In the Japanese standard manual, the cutoff values are defined according to percentile values corresponding to “average” (<1 SD), “high” (1–2 SD), and “very high” (>2 SD) for each age group of infants.

### 4.4. Statistics

In the analysis of BMs during sleep, we observed that the frequency of BMs followed a normal distribution prior to the analysis. We compared the average BMs during the first 3 h of sleep to those in the last 3 h using a *t*-test. Multiple linear regression analyses were employed to investigate the statistical impact of lifestyle habits on BMs and sleep latency. Variables related to lifestyle habits included in the multivariate models were selected based on our previous report [20]. Several models were tested using different combinations of these variables to evaluate statistical fit, interpretability, and consistency with established sleep science. Given the strong correlations between variables related to sensory traits, they were not included simultaneously in a single multiple regression model. The final model was selected based on its overall performance and alignment with theoretical expectations.

In this study, we coded lifestyle habits related to sleep, including breakfast consumption, nap end time, active play, and screen time. This coding was based on findings by Murata et al. [20], which showed that in the group with poor sleep hygiene, there were significantly more children who had “not much” breakfast (coded as 0), had a nap end time after 3:00 PM (0), had no morning activity (0), and had screen time exceeding 60 min (0). Therefore, these items were coded using a binary system of 0 and 1. To assess collinearity in the multiple regression analysis, we examined correlation coefficients among independent variables and confirmed that no major collinearity was present.

Additionally, we conducted correlation analysis to examine the relationship between BMs, sleep latency, and sensory processing patterns. Pearson’s correlation coefficient was utilized for oral sensory processing because it followed a normal distribution, whereas Spearman’s rank correlation coefficient was employed for auditory, visual, tactile, and vestibular sensory processing, as these variables did not meet the assumption of normality.

Statistical analyses were carried out using IBM SPSS Version 29.0 (IBM Corp., Armonk, NY, USA) and STATA/SE Version 17.0 (StataCorp LLC, College Station, TX, USA). All significant probability levels were set at *p* < 0.05.

## 5. Conclusions

Our findings suggest that lifestyle habits and sensory processing patterns may influence sleep onset and the first 3 h of sleep. Thus, the importance of creating a conducive sleep environment, limiting media use before bedtime, and encouraging calm bedtime routines should not be overlooked.

## Figures and Tables

**Table 1:** An overview of the sleep, sleep-related habits, and sensory processing characteristics of participants.

Toddler Demographics (*n* = 58)	
Age in months, mean ± SD	22.0 ± 2.0
Sex, male, *n* (%)	30 (51.7%)
Sleep onset, mean ± SD	9:24 PM ± 0:48
Sleep end, mean ± SD	6:49 AM ± 0:40
Total sleep time (min), mean ± SD	564.8 ± 43.9
Sleep latency (min), mean ± SD	33.3 ± 16.3
Net sleep time (min), mean ± SD	397.6 ± 65.1
Sleep efficiency (%), mean ± SD	64.5 ± 8.5
Consuming a substantial breakfast, *n* (%)	42 (72.4%)
Play outside in the morning, *n* (%)	30 (51.7%)
Nap end time, mean ± SD	3:17 PM ± 1:08
Daily screen time (min), mean ± SD	92.0 ± 69.0
Screen time of 1 h before bedtime, *n* (%)	10 (17.2%)
Amounts of BMs	mean ± SD
BMs 1 h before bedtime	6.12 ± 2.17
Total sleep BMs	0.43 ± 0.15
First 3 h BMs	0.29 ± 0.13
Last 3 h BMs	0.48 ± 0.16
Sensory processing scores	mean ± SD (normal range)
Auditory sensory	17.0 ± 4.0 (13–21)
Visual sensory	18.0 ± 3.0 (13–20)
Tactile sensory	29.0 ± 8.0 (21–36)
Vestibular sensory	15.0 ± 3.0 (12–17)
Oral sensory	16.0 ± 5.0 (10–18)

**Table 2:** The results of the multiple regression analysis of lifestyle factors on sleep latency.

		Sleep Latency (Time) (Adjusted R² = 0.22)			
		Coefficient	*p*	95% CI	
(*n* = 58)				Lower	Upper
Consuming a substantial breakfast		−7.43	0.089	−16.0	1.18
Playing outside in the morning		−4.50	0.245	−12.2	3.19
Nap end time		2.43	0.537	−5.42	10.3
Daily screen time		−0.66	0.875	−8.94	7.63
before bedtime	Screen time	12.7	0.016 *	−2.49	22.8
	BMs	2.86	0.003 *	1.05	4.67

* *p* < 0.05.

**Table 3:** The results of a multiple regression analysis of BMs during the first 3 h and the last 3 h in relation to various lifestyle factors.

		First 3 h BMs (Adjusted R² = 0.16)				Last 3 h BMs (Adjusted R² = −0.005)			
		Coefficient	*p*	95% CI		Coefficient	*p*	95% CI	
(n = 58)				Lower	Upper			Lower	Upper
Consuming a substantial breakfast		−0.096	0.009 **	−0.17	−0.03	−0.097	0.317	−1.50	0.05
Playing outside in the morning		0.048	0.129	−0.01	0.11	0.021	0.634	−0.68	0.11
Nap end time		−0.066	0.046 *	−0.13	−0.00	−0.014	−0.760	−0.10	0.54
Daily screen time		−0.008	0.808	−0.08	−0.06	0.087	0.075	−0.01	0.18
before bedtime	Screen time	0.065	0.126	−0.02	0.15	0.012	0.831	−0.11	0.13
	BMs	0.004	0.564	−0.01	−0.03	0.010	0.326	−0.01	0.03

*p < 0.05, ** *p* < 0.01.

**Table 4:** The correlation between sleep latency and BMs during the first 3 h and the last 3 h and the sensory processing scores.

	Sleep Latency	First 3 h BMs	Last 3 h BMs
Auditory sensory	0.167	0.291 *	0.039
Visual sensory	−0.276 *	−0.062	−0.008
Tactile sensory	0.040	0.031	0.074
Vestibular sensory	0.064	0.131	0.147
Oral sensory	0.204	0.299 *	0.137

Pearson correlation was used for oral sensory scores, and Spearman correlation was used for the other sensory scores because of non-normal distribution. * *p* < 0.05.

## Data Availability

The data supporting the reported results in this study are not available because of privacy restrictions.

## Abbreviations

The following abbreviations are used in this manuscript:

SD	standard deviation
BMs	body movements
Total sleep BMs	BMs during overnight sleep
First 3 h BMs	BMs during the first 3 h of sleep from sleep onset
Last 3 h BMs	BMs during the last 3 h of sleep until the end of sleep
